# Effectiveness of alcohol-based hand disinfectants in a public administration: Impact on health and work performance related to acute respiratory symptoms and diarrhoea

**DOI:** 10.1186/1471-2334-10-250

**Published:** 2010-08-24

**Authors:** Nils-Olaf Hübner, Claudia Hübner, Michael Wodny, Günter Kampf, Axel Kramer

**Affiliations:** 1 Institute of Hygiene and Environmental Medicine, Walter-Rathenau-Straße 49 A 17487 Greifswald, Germany; 2 Institute of Biometrics and Medical Informatics, Walther-Rathenau-Str. 48 17475 Greifswald, Germany; 3 Bode Chemie GmbH, Scientific Affairs, Melanchthonstrasse 27, 22525 Hamburg, Germany

## Abstract

**Background:**

The economical impact of absenteeism and reduced productivity due to acute infectious respiratory and gastrointestinal disease is normally not in the focus of surveillance systems and may therefore be underestimated. However, large community studies in Europe and USA have shown that communicable diseases have a great impact on morbidity and lead to millions of lost days at work, school and university each year. Hand disinfection is acknowledged as key element for infection control, but its effect in open, work place settings is unclear.

**Methods:**

Our study involved a prospective, controlled, intervention-control group design to assess the epidemiological and economical impact of alcohol-based hand disinfectants use at work place. Volunteers in public administrations in the municipality of the city of Greifswald were randomized in two groups. Participants in the intervention group were provided with alcoholic hand disinfection, the control group was unchanged. Respiratory and gastrointestinal symptoms and days of work were recorded based on a monthly questionnaire over one year. On the whole, 1230 person months were evaluated.

**Results:**

Hand disinfection reduced the number of episodes of illness for the majority of the registered symptoms. This effect became statistically significant for common cold (OR = 0.35 [0.17 - 0.71], p = 0.003), fever (OR = 0.38 [0.14-0.99], p = 0.035) and coughing (OR = 0.45 [0.22 - 0.91], p = 0.02). Participants in the intervention group reported less days ill for most symptoms assessed, e.g. colds (2.07 vs. 2.78%, p = 0.008), fever (0.25 vs. 0.31%, p = 0.037) and cough (1.85 vs. 2.00%, p = 0.024). For diarrhoea, the odds ratio for being absent became statistically significant too (0.11 (CI 0.01 - 0.93).

**Conclusion:**

Hand disinfection can easily be introduced and maintained outside clinical settings as part of the daily hand hygiene. Therefore it appears as an interesting, cost-efficient method within the scope of company health support programmes.

**Trial registration number:**

ISRCTN96340690

## Background

Absenteeism and reduced productivity due to communicable illness, in particular acute infectious respiratory and gastrointestinal disease, are a major problem for national economies worldwide [1-5]. But because acute upper respiratory infections ("common cold") or mild cases of infectious gastrointestinal illness have a very low mortality, are in most cases short timed and self limiting they are assumed to be less costly per case than chronic conditions. Therefore, their economical impact is often underestimated and they are normally not in the focus of surveillance systems. However, large community studies in Europe and in USA have shown that communicable diseases have a great impact on morbidity and lead to millions of lost days at work, school and university each year [6]. Fendrick et al., for example, estimated the total economic impact of non-influenza-related viral respiratory tract infections in the USA with $40 billion annually. Due to their high prevalence in working-age groups they have the potential to cause substantial health-related productivity losses [7]. This results not only in missed work time and caregiver absenteeism, but in high on-the-job productivity loss due to impaired work performance, too [7]. It has been shown that acute upper respiratory illnesses can reduce one's effectiveness at work, including subjective alertness and psychomotor [5,8-15]. Economical investigations have proven that besides direct illness costs, indirect costs due to missed work time (absenteeism), caregiver absenteeism and on-the-job productivity loss, accounts for the biggest part of expenses caused by acute communicable illness [7].

While no specific protection exists against these diseases, personal hygiene, especially hand hygiene, has been acknowledged as a key element to prevent the spread in the community [16]. The efficacy of hand disinfection in medical facilities has been demonstrated a number of times [17-24]. Studies assessing the effect of the implementation of hand-hygiene regiments in non-clinical settings such as children day cares, school and university campuses or military training camps have also shown significant reductions in communicable illness and absenteeism rates [25-28]. The effectiveness of hand disinfection in open community work place settings like a public administration however has not been assessed so far. Our study provides an initial investigation of the impact of alcohol-based hand disinfectant use at work place by assessing illness rates due to common cold and diarrhoea. We furthermore estimate the economic benefits to be expected by further application of hand disinfection at work.

## Methods

### Enrolment of participants and data collection

**Figure 1:**
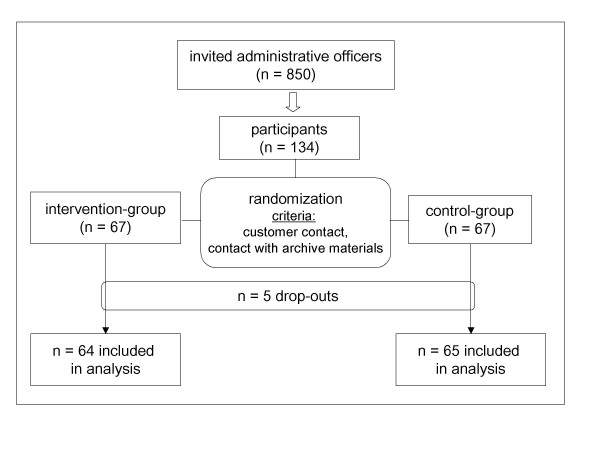
**Flow chart showing randomization of participants**.

This study involved a prospective, controlled, randomized design. We recruited employees from the administration of the Ernst-Moritz-Arndt University Greifswald, the municipality of Greifswald and the state of Mecklenburg-Pomerania, for the study. All administrative officers, who do not already apply hand disinfection at work, were considered for participation and got invited by e-mail or mail (n = 850). 134 persons declared their written consent to participate and completed a pre-study survey with demographic, social, health and work related questions to provide data for randomization. Participants were randomized in control (n = 67) and intervention (n = 67) group based on the frequency of customer contact and work with paper documents, especially archive materials (Figure 1, Table 1). Based on the existing literature, we hypothesised, that these factors have the most relevant impact on the transmission of pathogenic organisms in administrations and therefore set as covariates [29-40]. Employees that already used hand disinfectants at work were excluded from the study.

**Table 1:** Randomisation and distribution of evaluable participants to groups (total(control/intervention))

Customer contact work with archive material	frequently	occasionally	Seldom or never	Σ
Daily	24 (11/13)	20 (10/10)	16 (7/9)	60 (28/32)

seldom or never	35 (19/16)	22 (11/11)	12 (7/5)	69 (37/32)

Σ	59 (30/29)	42 (21/21)	28 (14/14)	129 (65/64)

Two alcohol based hand rubs were used in this study: Amphisept E^® ^(Bode Chemie, Hamburg, Germany) is an ethanol (80% w/w) based formula and has antibacterial, antifungal and limited virus inactivating activity. Participants facing skin problems (increased dryness, redness, itching, reported by participants) were provided with Sterillium^® ^(Bode Chemie, Hamburg, Germany) which is based on 2-propanol (45% w/w), 1-propanol (30% w/w) and mecetronium etilsulfate (0.2% w/w), is known to have a refatting effect and has activity against bacteria, fungi and enveloped viruses [41-43]. Both products fulfil the requirements of the DIN EN 12791 (surgical hand disinfection) and DIN EN 1500 (hygienic hand disinfection) and can therefore be seen as equally effective [44,45]. Rubs were provided in 500 ml bottles for desktop use to ensure minimal effort for use. For skin care, all participants in the intervention group were provided with hand cream care Baktolan^® ^balm, water-in-oil-emulsion with no non-antibacterial properties (Bode Chemie, Hamburg, Germany).

Participants in the intervention group were instructed to use as much product as needed for complete wetting of the hands (at least 3 ml or a palmful) of hand rub to ensure in accordance with the DIN EN 1500 (standard procedure) and advised to use it at least five times daily, especially after toilet use, blowing nose, before eating and after contact with ill colleagues, customers, and archive material [44].

Participants were provided with hand rub as needed and instructed to use the hand rub only at work, while hand hygiene at home was not changed.

Hand hygiene remained unchanged in the control group. During the study, close contact was maintained with all participants. This included individual contact (at least monthly) either personally or by phone or e-mail. All participants were provided with contact details and could contact the study management at any time.

The study was started in March 2005 and lasted until April 2006. Surveys were sent to participants of both groups collecting data on illness symptoms (common cold, sinusitis, sore throat, fever, cough, bronchitis, pneumonia, influenza, diarrhoea) and associated absenteeism at the end of every month. Definitions of symptoms were given to the participants as part of the individual information at the beginning of the study. While most symptoms are quite self-explanatory, "influenza" and "pneumonia" are specific diagnoses that were asked state when confirmed by professional dia-gnosis only. Similarly, (self-)diagnosis of "fever" required objective measurement with a thermometer. Furthermore, compliance with hand hygiene measures was queried [46,47]. Test persons reported illness (ill but not absent) and absenteeism (absent from work due to illness) days per month separately for each symptom. Appearance of at least one day ill was counted as an illness episode for the current month. There was no distinction made between the number of episodes within a month. After 12 months, participants filled out a post-study survey to assess post-intervention compliance with hand hygiene [48]. Ethical approval for the study was obtained from the ethics committee of the University of Greifswald, Germany (Reg. No.: BB 02/10) and registered with the ISRCTN-register (Reg. No.: ISRCTN96340690.

### Statistical analysis

All data from surveys was collected in a database (Microsoft Access 2003, Microsoft Corporation, Redmond, WA, USA) and analysed in SPSS 15 (SPSS Chicago Inc.).

To analyse the number of independent episodes of illness or absence, the number of months with and without symptoms or absence was determined, respectively. The odds ratio (OR) and confidence intervals between the two groups were then calculated and the Χ^2^- Test used to detect statistically significant differences between groups (significance level p = 0.05).

To test for statistically significant differences in the total number of days absent or ill data were analysed using multivariate tests. Because data were shown to break the assumptions for parametric procedures, univariate and multivariate analysis of variance (ANOVA/MANOVA) or covariance (ANCOVA/MANCOVA) were not applicable. Therefore we used the non-parametric approach of Puri and Sen's L-statistic to analyse data [49,50]. Frankly, the L-statistic, as other non-parametric test like the Wilcoxon-test, first changes data in each variable to ranks by assigning the rank of 1 to the lowest (or highest score) 2 to the next and so on up to the number of participants. Thereafter uni- or multivariate tests are performed using the ranked data. From the tests summary table, r^2 ^as the proportion of true variance (SS_regression_/SS_total_) is calculated and used to calculate L using the Equation (N = number of participants):

L=(N−1)∗r²

The L-statistic is then compared to Χ^2 ^with pq-degrees of freedom (p = number of independent variables, q = number of dependent variables). This method is robust against violations of the described assumptions and has been shown to be superior to its parametric pendants in terms of power and type one error, when assumptions are broken [51].

At first, a non-parametric MANCOVA using the L-Statistic was used to test for global differences for all symptoms and associated days absent as omnibus test. Special effects were then determined using non-parametric ANCOVA. Differences in the number of days absent were only assessed if differences for the associated days ill were significant [52].

## Results

From 850 employees asked to participate, 134 could be included in the study and data from 129 participants (64 in the intervention and 65 in the control group) were finally analyzable. During the trial, 10 participants (15.6%) switched from Amphisept E to Sterillium.

Persons (n = 5) who did not return at least one evaluable survey were excluded from the analysis. Every returned survey was counted as one person month. Overall, datasets of 1230 person months (79.46% of total possible follow-up surveys) were collected.

Compliance with hand hygiene was high during the study. Mean hand disinfection frequency reported was more than 5 times daily in 19%, 3-5 times daily in 59.8%, and 1-2 times daily in 20.5% of the person month. In only 0.7% of person month an average frequency of hand disinfection lower than 1 per day was reported. There was no statistically significant change in compliance during the study (Χ^2^-test, p = 0,387) [46,47].

### Data from pre-study survey

Randomization in both groups was based on frequency of customer contact and contact with archive materials. According to the pre-study survey, participants were allocated to one of six groups, which were then randomly split by half into control and intervention group and as equally as possible (table 1). From all participants 45.7% declared to have customer contact frequently and 46.5% to have contact to archive materials daily

There were no significant differences in the mean age, size of household, number of children, smoking, exercise frequency or means of transportation to work. (table 2). The difference in the distribution of women and men between the groups was unintentional.

**Table 2:** Baseline demographic data of evaluable participants

criteria		intervention group (n = 64)	control group (n = 65)	p =
sex	female	60	51	0.012
		
	male	4	14	

mean age		43.6	45.6	0.257

size of household	1-person	7	9	0.561
		
	2-person	24	25	
		
	3-person	19	12	
		
	4-person	11	14	
		
	5(+)-person	3	5	

number of children (< 16 years)	no children	46	51	0.140
		
	1 child	15	8	
		
	2 children	2	6	
		
	3 children	1	0	

smoker	yes	12	17	0.314
		
	no	52	48	

regularly participate in sport exercises	yes	32	40	0.187
		
	no	32	25	

means of transportation to work	walking	10	7	0.830
		
	bike	23	23	
		
	car	30	31	
		
	others	1	4	

### Effect on the number of single episodes of illness or absence

Odds for being (ever) ill or absent and odds ratios (OR) between groups are presented in table 3 and 4. Frankly, hand disinfection lowered the odds to get ill with the exception of sinusitis and bronchitis. This effect became statistically significant for common cold (OR = 0.35 [±95% Confidence Interval (CI):0.17 - 0.71], p = 0.003), fever (OR = 0.38 [CI: 0.14 - 0.99], p = 0.035) and coughing (OR = 0.45 [CI: 0.22 - 0.91], p = 0.02). For absenteeism, this trend continued, with the addition that the difference became statistically significant for diarrhoea too (OR = 0.11 [CI: 0.01 - 0.93], p = 0.017). As in table 3, a difference favouring the control group was seen for bronchitis, but confidence intervals touched an OR of 1 (Table 4).

**Table 3:** Odds and OR for being ill

Symptom		Control			Intervention		OR (± 95%CI)
		
	no	yes	odds	no	yes	odds	
common cold	21	44	2.10	37	27	0.73	0.35 (0.17 - 0.71)*

Sinusitis	61	4	0.07	57	7	0.12	1.87 (0.52 - 6.74)

sore throat	31	34	1.10	38	26	0.68	0.62 (0.31 - 1.25)

Fever	49	16	0.33	57	7	0.12	0.38 (0.14 - 0.99)*

Coughing	30	35	1.17	42	22	0.52	0.45 (0.22 - 0.91)*

Bronchitis	60	5	0.08	55	9	0.16	1.96 (0.62 - 6.22)

Pneumonia	62	3	0.05	61	3	0.05	0.96 (0.96 - 1.01)

Influenza	62	3	0.05	61	3	0.05	1.02 (0.20 - 5.23)

Diarrhoea	50	15	0.30	56	8	0.14	0.48 (0.19 - 1.22)

*statistically significant result (Χ^2^-Test, p < 0.05)

**Table 4:** Odds and OR for being absent

Symptom		Control			Intervention		OR (± 95%CI)
		
	no	yes	odds	no	yes	odds	
common cold	46	19	0.41	53	11	0.21	0.50 (0.22 - 1.17)

sinusitis	63	2	0.03	58	6	0.10	3.26 (0.63 - 16.79)

sore throat	52	13	0.25	52	12	0.23	0.92 (0.39 - 2.21)

fever	55	10	0.18	58	6	0.10	0.57 (0.19 - 1.67)

coughing	51	14	0.27	52	12	0.23	0.84 (0.36 - 1.99)

bronchitis	63	2	0.03	55	9	0.16	5.16 (1.07 - 24.88)*

pneumonia	64	1	0.02	64	0	0.00	0.985 (0.96 - 1.02)

influenza	62	3	0.05	61	3	0.05	1.02 (0.20 - 5.23)

diarrhoea	57	8	0.14	63	1	0.02	0.11 (0.01 - 0.93)*

*statistically significant result (Χ^2^-Test, p < 0.05)

### Effect on the total number of days absent or ill

Nonparametric analysis of co-variance revealed a significant difference in days ill between groups (MANCOVA, p = 0.01). Significantly fewer days with symptoms of colds, fever and cough were reported by the intervention group. The strongest effect was identified for colds (p = 0.008). Detailed Χ^2^-test statistics, degrees of freedom and p-values are presented in Table 5.

**Table 5:** Percentage of days ill and Test statistics for MANCOVA

symptom	control	intervention	difference	Χ^2^-test statistics	p-values
common cold	2.78	2.07	- 0.71	7.040	0.008 *

sinusitis	0.12	0.34	+ 0.22	1.024	0.312

sore throat	1.53	1.34	- 0.19	0.640	0.424

fever	0.31	0.25	- 0.05	4.352	0.037 *

cough	2.00	1.85	- 0.14	5.120	0.024 *

bronchitis	0.20	0.39	+ 0.19	1.408	0.235

pneumonia	0.08	0.00	- 0.08	1.152	0.283

influenza	0.12	0.13	- 0.01	0.000	1.000

diarrhoea	0.92	0.11	- 0.82	3.200	0.074

*statistically significant result (p < 0.05)

For colds, fever and cough a follow-up analysis of days absent was performed. While there was a trend in favour of the intervention group, the difference did not become statistically significant (Table 6).

**Table 6:** Percentage and Test statistics for MANCOVA of days absent

symptom	Control	Test	Difference	Χ^2^-test statistics	p-values
common cold	0.372	0.365	- 0.007	1.920	0.166

fever	0.158	0.225	+ 0.067	0.896	0.344

cough	0.372	0.416	+ 0.044	0.256	0.613

*statistically significant result (p < 0.05)

## Discussion

This study is one of the first investigations on the effectiveness of hand disinfection with alcoholic rubs in a public administration as an example of an open, non-clinical setting with working adults. Our results confirm the findings from other authors, that hand disinfection has preventive effects against acute respiratory and gastrointestinal infections [53-59]. Data were analysed under the aspects of the effect on the number of single episodes of illness or absence per year and the effect on the total number of days absent or ill.

It could be shown, that hand disinfection has a reducing influence on the number of episodes of illness for the majority of the registered symptoms, with the strongest effects for common cold, coughing, fever and diarrhoea. On average, participants of the intervention group who used alcoholic hand disinfection at their workplace declared less illness episodes and therefore more symptom-free months during the year in comparison with the control group. This confirms data from other authors that the use of hand disinfection leads to interruptions of transmission chains which results in fewer illness episodes [55,59-63].

The analysis of the total number of days ill for most symptoms over the year proved similar reducing effects for hand disinfection. As expected, the highest infection rates for respiratory symptoms were measured during the winter months [64,65]. For that reason we observed the highest reduction effects in the intervention group also during winter. In contrast, we saw no seasonal peaks in the incidence of diarrhoea and so effects of hand sanitizer use were quite similar every month. Overall, a decline of days ill could was seen for most symptoms compared to the control group.

In both analyses (days/episodes) the impact on absenteeism was lower than the effect on the total of days ill. This confirms that not every case of illness with banal diseases does necessarily lead to a sick note. Rather in spite of taking illness absenteeism, employees continue working if possible, but work performance is restricted and many times insufficient. This results in an often underestimated on-the-job productivity loss.

For office work, on-the-job productivity loss is especially difficult to assess, due to the high complexity of work and the tasks performed.

In most studies on the topic, assessments of the impact on health-related productivity loss base on questionnaires on subjective items like alertness, psychomotor functioning or reaction [66]. Taken together, these studies imply the importance of impaired productivity for understanding the indirect costs associated with these illnesses. However, it is difficult to calculate explicit illness cost with these data. Nevertheless the amount of sick notes or the number of days off work may not be taken as a sole factor for the measurement of indirect illness costs. Additionally, other aspects which are not that evident and often hardly measurable like the on-the-job productivity loss have to be strongly considered. Our results confirm that there is no fixed correlation between days ill and days absent or between missed work time and on-the-job productivity loss, respectively. Hand disinfectant use reduces primarily the number of illness days which leads to less on-the-job productivity loss and consequently to a decrease in indirect illness costs. While one would expect that hand disinfection should reduce the number of days of work likewise the days ill, our trial lacked the power to show this effect.

Our study has several limitations. Only 16% of invited persons could be included in the study. Due to this, possible effects of hand hygiene are potentially underestimated. Effects of symptoms that are relatively rare but often associated with a chronic disposition or take a longer time to heal like sinusitis or bronchitis are hard to interpret on the other hand, because a single episode can have an ordinate effect. Assessment of days ill or absent as well as single episodes was based on monthly surveys. While this approach has been used by various authors [59,61,63], a more refined assessment could have revealed smaller differences. Moreover, with a more detailed questionnaire, perceiving not only the symptom but the markedness of the symptom, possible effects on productivity loss could be more precisely estimated.

Further research should be focussed on the question how on-the-job productivity losses could be assessed more clearly, allowing exact calculations on the cost-effectiveness of hand hygiene programmes and on the relationship between the frequency of hand hygiene and symptoms. Still, our data supports the results from other studies, that hands play a key role in the transfer of community-acquired viral and bacterial infection.

## Conclusion

We were able to demonstrate that hand disinfection can easily be introduced and maintained outside clinical settings as a part of the daily hand hygiene [46,47]. Therefore it appears as an interesting, and probably cost-efficient method within the scope of company health support programmes.

## Competing interests

The authors declare a financial competing interest: GK is employed by Bode Chemie GmbH, Hamburg, Germany. NOH and AK received financial support for research from Bode Chemie in the past. All other authors declare no conflict of interest.

## Authors' contributions

NOH had the idea for the study and planned and supervised the trial, analyzed and interpreted the data and drafted the manuscript. CH participated in the design of the study, conducted the trial, helped to analyze and interpret the data and to draft the manuscript. MW helped to analyze and interpret the data and to draft the manuscript. GK helped to draft and revise the manuscript. AK participated in the study design and coordination, and helped to interpret the data and to draft the manuscript.

All authors have been involved in drafting the manuscript or revising it critically for important intellectual content and have read and approved the final manuscript.

## Pre-publication history

The pre-publication history for this paper can be accessed here:

http://www.biomedcentral.com/1471-2334/10/250/prepub
